# Geographical distribution-based differentiation of cultivated *Angelica dahurica*, exploring the relationship between the secretory tract and the quality

**DOI:** 10.1038/s41598-023-48497-4

**Published:** 2023-12-08

**Authors:** Qinghua Wu, Lan Jiang, Yuhang Yan, Qi Yan, Xinglong Zhu, Jiaxu Zhang, Chengfeng Huang, Tao Zhou, Chaoxiang Ren, Feiyan Wen, Jin Pei

**Affiliations:** 1 State Key Laboratory of Southwestern Chinese Medicine Resources, Chengdu, 611137 China; 2 School of Pharmacy, Chengdu University of Traditional Chinese Medicine, Chengdu, 611137 China

**Keywords:** Plant sciences, Plant biotechnology

## Abstract

Based on geographical distribution, cultivated Chinese *Angelica dahurica* has been divided into *Angelica dahurica* cv. ‘Hangbaizhi’ (HBZ) and *Angelica dahurica* cv. ‘Qibaizhi’ (QBZ). Long-term geographical isolation has led to significant quality differences between them*.* The secretory structure in medicinal plants, as a place for accumulating effective constituents and information transmission to the environment, links the environment with the quality of medicinal materials. However, the secretory tract differences between HBZ and QBZ has not been revealed. This study aimed to explore the relationship between the secretory tract and the quality of two kinds of *A. dahurica.* Root samples were collected at seven development phases. High-Performance Liquid Chromatography (HPLC) and Desorption Electrospray Ionization Mass Spectrometry Imaging (DESI-MSI) were used for the content determination and spatial location of coumarins. Paraffin section was used to observe and localize the root secretory tract. Origin, CaseViewer, and HDI software were used for data analysis and image processing. The results showed that compared to QBZ, HBZ, with better quality, has a larger area of root secretory tracts. Hence, the root secretory tract can be included in the quality evaluation indicators of *A. dahurica*. Additionally, DESI-MSI technology was used for the first time to elucidate the temporal and spatial distribution of coumarin components in *A. dahurica* root tissues. This study provides a theoretical basis for the quality evaluation and breeding of improved varieties of *A. dahurica* and references the DESI-MSI technology used to analyze the metabolic differences of various compounds, including coumarin and volatile oil, in different tissue parts of *A. dahurica*.

## Introduction

*Angelica dahurica* has been used as a food and medicine herb in China for over 1000 years. *A. dahurica* extracts have pharmacological effects, including anti-inflammatory, analgesic, antispasmodic, anti-tumor, antiviral, antihypertensive, and anti-sugar^1–3^. Each Chinese Pharmacopoeia edition records that *A. dahurica* is derived from the dried root of *A. dahurica* (Fisch. ex Hoffm) Benth.et Hook. f. or *A. dahurica* (Fisch. ex Hoffm) Benth.et Hook. f. var. formosana (Boiss.) Shan et Yuan^4^. However, disputes exist regarding the relationship between various cultivated varieties of *A. dahurica*. *A. dahurica* artificial cultivars have yielded four mainstream commercial drugs including Qibaizhi, Yubaizhi, Hangbaizhi, and Chuanbaizhi^5^. Yuan Cq et al. identified and classified the original *A. dahurica* plants, concluding that Hangbaizhi and Chuanbaizhi originated from *A. dahurica* cv. ‘Hangbaizhi’ (HBZ), while Qibaizhi and Yubaizhi originated from *A. dahurica* cv. ‘Qibaizhi’^6^ (QBZ). Xie Zw and Huang Lq reported that the four major commodities of *A. dahurica* originated from *A. dahurica* cv. ‘Hangbaizhi’^7,8^. However, other studies reported that the four major commodities of *A. dahurica* all originated from *A. dahurica*^9,10^. Most researchers have recently accepted the first viewpoint that the original *Angelica dahurica* is divided into two categories based on their geographical distribution in the north and south^11^. Evidence revealed significant quality and low genetic similarity differences between the two varieties of *A. dahurica*^12–14^*.*

The secretory tract is a typical feature of *A. dahurica*’s microstructure as a representative plant of the Apiaceae family. The secretory tract of *A. dahurica* is the accumulation site of its effective components, such as coumarin and volatile oil^15,16^. Simultaneously, some toxic coumarin components accumulate in the secretory tract as a defense against herbivorous and pathogenic organisms^17,18^. According to Roshcina, secretory activity in plants expresses the ability to exchange substances and energy with the environment, and the secretory structure is influenced by the environment^19^. A study on *Aeollanthus suaveolens’*s leaves anatomy displayed that the secretory cavities of *Aeollanthus suaveolens’*s leaves exposed to dry and sunlight were significantly larger than those exposed to damp and cool conditions^20^. Studies on Pinaceae xylem anatomy revealed that extreme environmental conditions, including high temperatures, aridity, and cold winters, were associated with higher abundance and greater sizes of resin canals^21^. Based on this, it is reasonable to assume that the climate and environmental variables between the north and south lead to different stable genetic secretory structure formation in Qibaizhi and Hangbaizhi, thereby affecting the coumarin component accumulation and leading to the formation of stable genetic germplasm differences of Qibaizhi and Hangbaizhi. Regrettably, there is no relevant literature has been reported.

Mass spectrometry imaging (MSI) is a molecular imaging technique that combines mass spectrometry and image visualization to enable a qualitative, quantitative, and localized analysis of thousands of metabolites in biological tissues^22^. It is widely used in traditional Chinese medicine (TCM) research because it accurately characterizes the spatial distribution of metabolites in medicinal plants and helps to infer the metabolite accumulation pattern^23,24^. The commonly used MSI techniques include matrix-assisted laser desorption ionization mass spectrometry imaging (MALDI-MSI), air flow assisted ionization mass spectrometry imaging (AFAI-MSI), secondary ion mass spectrometry imaging (SI-MSI), and desorption electrospray ionization mass spectrometry imaging (DESI-MSI)^25–27^. DESI-MSI is widely used in the research of difficult-to-slice TCM because it has a low cost, fewer sample preparation requirements, and no matrix assistance^28^. Although researchers have used the MALDI-MSI technique to analyze the spatial distribution of coumarin components in the main and lateral roots of *A. dahurica*^29^, this study did not correlate coumarins’ spatial distribution with their accumulation in the secretory tract.

In this study, we collected seeds of HBZ and QBZ, then planted them uniformly. The root samples were collected at seven different phenological stages to study the differences in the content and localization of coumarin components, as well as differences in the development and localization of root secretory canals, and finally to analyze the correlation between coumarin and the distribution coefficient of root secretory canal. This study is expected to provide a theoretical basis and reference for quality evaluation and improved variety selection of *A. dahurica.*

## Results

### The difference in coumarin content between the roots of HBZ and QBZ in seven periods

**Figure 1:**
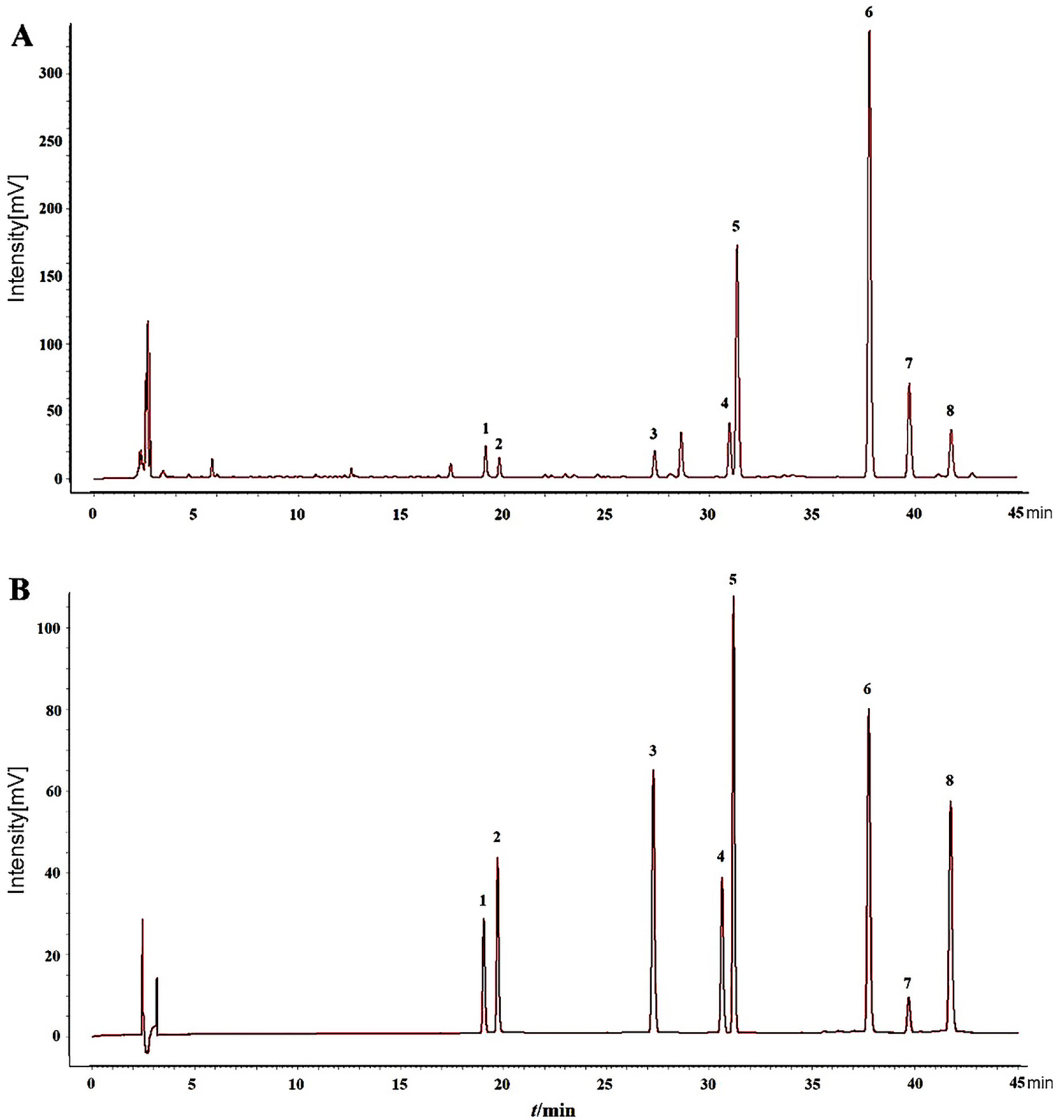
HPLC chromatograms of references and samples (**A** sample; **B** reference; 1: Oxypeucedanin hydrate; 2: Byakangelicin; 3: Bergapten; 4: Byakangelicol; 5: Oxypeucedanin; 6: Imperatorin; 7: Phellopterin; 8: Isoimperatorin).

**Figure 2:**
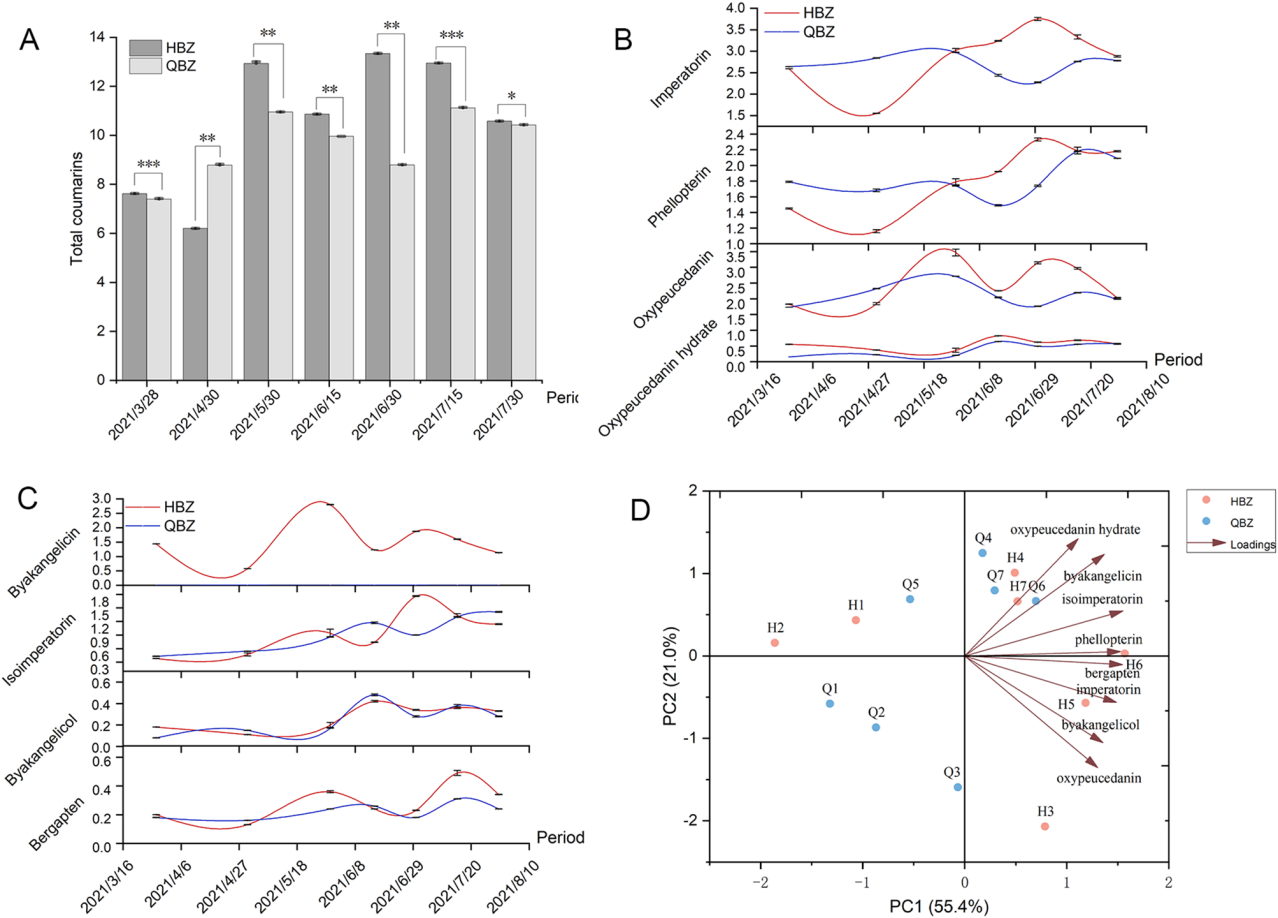
Dynamic changes of coumarin content in seven developmental stages of HBZ and QBZ, and PCA of HBZ and QBZ (**A** Total coumarins; **B** Content of oxypeucedanin hydrate, imperatorin, phellopterin, and bergapten; **C** Content of isoimperatorin, oxypeucedanin, byakangelicol, and byakangelicin; **D** PCA of HBZ and QBZ. Data are means ± SD. *p < 0.05; **p < 0.01; ***p < 0.001).

The chromatographic peaks of eight coumarin components were well separated under the set chromatographic conditions (Fig. 1). Tables S1–S5 display the parameters of linearity, precision, stability, repeatability, and recovery test. Figure 2 and Table S6 present the content changes of eight coumarin components in two varieties of *A. dahurica* at seven development stages. Figure 2 reveals that the eight coumarin components of the two types of *A. dahurica* displayed dynamic changes in the seven periods with an overall increasing trend. The mass fraction of imperatorin should be more than 0.08% based on the dried product as a quality control component specified in the 2020 edition of Chinese Pharmacopoeia. In our experiment, all 14 measured samples conformed to the regulations, and the imperatorin content was the highest of any period compared to other coumarin components. The eight coumarins can be roughly divided into three categories by comparing HBZ with QBZ from the perspective of the composition. In the first category, the overall change of imperatorin, phellopterin, and oxypeucedanin content exhibited an “S” pattern. They were lower in HBZ than in QBZ in the early developmental stage (late March and April). However, HBZ was higher than QBZ and had been in the lead composition in the late growth stage. In the second category, the overall change of isoimperatorin, bergapten, byakangelicol, and byakangelicin content exhibited an “M” pattern. They were more in HBZ than in QBZ in late May and late June, and HBZ had been in a dominant position until late July. In the third category, HBZ contained more oxypeucedanin hydrate than QBZ in seven periods. From the perspective of the development period, the total content of eight coumarins in HBZ peaked in late June, ranked second in the middle of July, and peaked in the middle of July in QBZ.

### Principal component analysis (PCA) of eight coumarins in HBZ and QBZ

PCA of eight coumarin components of two varieties of *A. dahurica* was performed using SMICA software. Two principal components were extracted. The contribution rates of the characteristic values were 55.4% and 21.0%, respectively. The cumulative contribution rate reached 76.4%, indicating that the two principal components can represent the internal quality of *A. dahurica*. Figure 2D presents the biplot of eight coumarin components of HBZ and QBZ. PC1 primarily reflected the information on isoimperatorin, byakangelicol, bergapten, imperatorin, byakangelicin, and phellopterin. PC2 mainly reported the information on oxypeucedanin and oxypeucedanin hydrate. The quality difference between the two varieties of *A. dahurica* was evaluated using the PCA comprehensive score of two principal components. The comprehensive score was calculated as the sum of the product of the principal component factor scores and the contribution rate of the eigenvalues (Table 1). The comprehensive score of H6 was the highest, followed by H5, and Q6 ranked third.

**Table 1:** The comprehensive quality score of *A. dahurica* samples.

Sample	PC1 score	PC2 score	Comprehensive score	Rank
H1	− 1.06717	0.43248	− 0.50039	11
H2	− 1.8607	0.15784	− 0.99768	14
H3	0.78783	− 2.07	0.001758	8
H4	0.49112	1.00693	0.483536	4
H5	1.18612	− 0.56896	0.537629	2
H6	1.56886	0.02822	0.875075	1
H7	0.51752	0.66186	0.425697	5
Q1	− 1.32177	− 0.58144	− 0.85436	13
Q2	− 0.86973	− 0.86773	− 0.66405	12
Q3	− 0.06719	− 1.59218	− 0.37158	10
Q4	0.17609	1.24777	0.359586	6
Q5	− 0.53504	0.68804	− 0.15192	9
Q6	0.69912	0.66314	0.526572	3
Q7	0.29492	0.79404	0.330134	7

### Characterization of coumarins in* A. dahurica* using DESI-MSI

**Figure 3:**
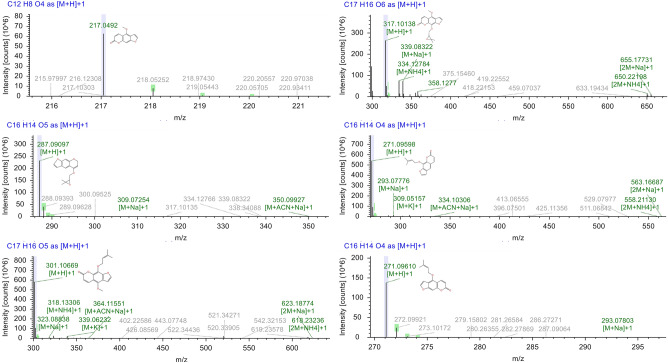
Primary mass spectrum of target coumarin.

Figure 3 illustrates the mass spectrum of the coumarin components in *A. dahurica* roots obtained in positive ion mode. The ions of m/z 317.1014, 287.0910, 271.0960, 301.1067, and 271.0970 were separately identified as byakangelicol [M + H]^+^, oxypeucedanin [M + H]^+^, imperatorin [M + H]^+^, phellopterin [M + H]^+^, and isoimperatorin [M + H]^+^, respectively. The errors are all less than 5 ppm. Table S7 summarizes the peak assignment of these identified ions. The fragment of oxypeucedanin hydrate bound to ions was not detected because it reacted with ethanol during mass spectrometry analysis and existed as a hydrogen loss peak, while byakangelicin underwent water loss during mass spectrometric detection, and its m/z was consistent with that of byakangelicol so that byakangelicin was discarded. Bergapten has extremely strong sublimation properties, causing component shifts during the detection process, which is not conducive to subsequent DESI-MSI detection.

### Spatial localization of coumarin compounds in HBZ and QBZ

**Figure 4:**
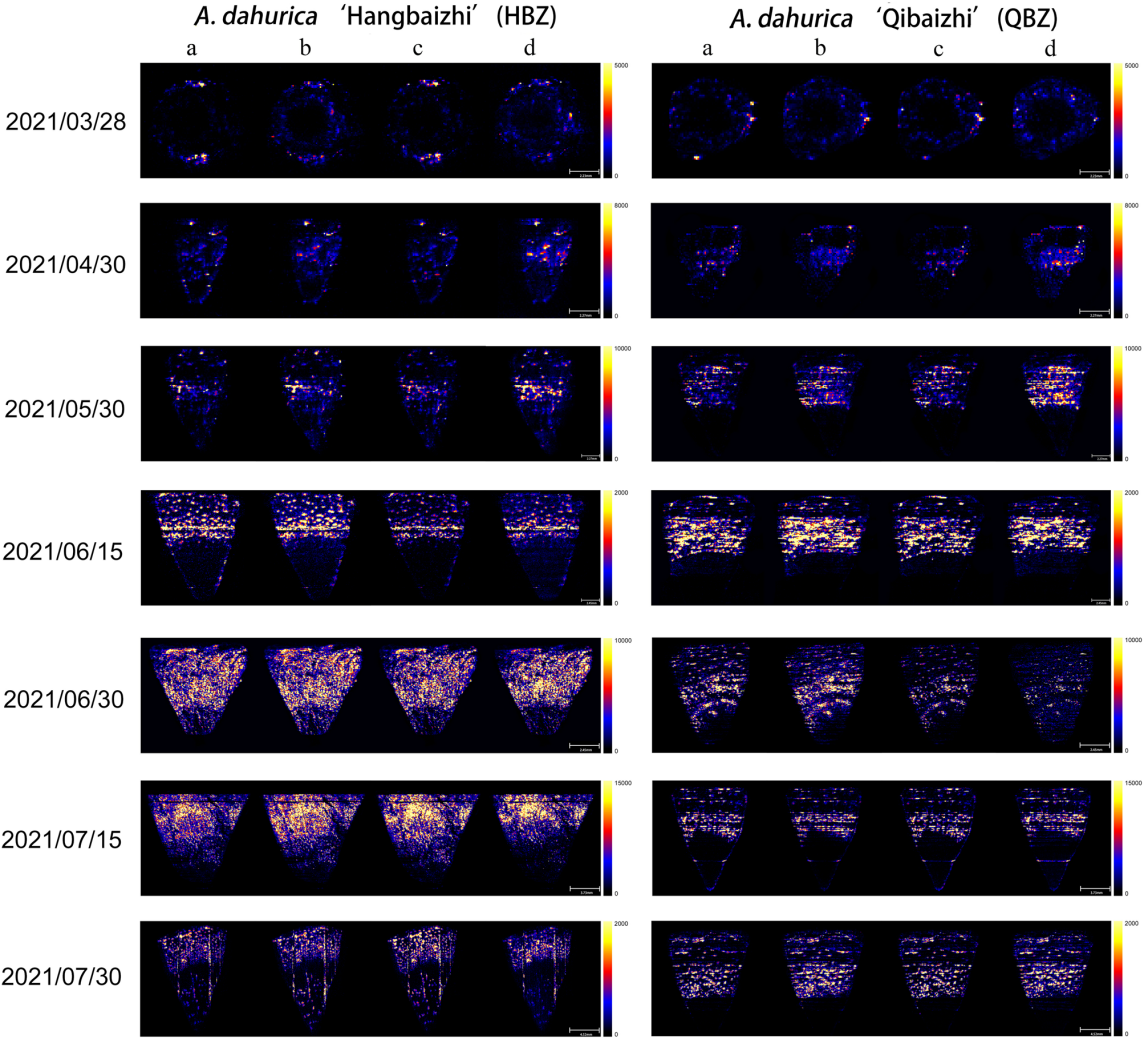
The spatial distribution of coumarins in the root sections of HBZ and QBZ (a: imperatorin and isoimperatorin; b: oxypeucedanin; c: phellopterin; d: byakangelicol; Scale bars: 2021/03/28: 2.23 mm; 2021/04/30, 2021/05/30: 2.27 mm; 2021/06/15, 2021/06/30: 2.45 mm; 2021/07/15: 3.73 mm; 2021/07/30: 4.52 mm).

Figure 4 depicts the spatial distribution of five coumarins in the root sections of HBZ and QBZ under different development stages. The spatial localization of five coumarin components in HBZ and QBZ roots is consistent. The five coumarin components of the two varieties of *A. dahurica* were distributed in the roots’ outer layer, namely, the cortex in late March. The five coumarin components of the two *A. dahurica* were distributed in the cortex and phloem from late April to late July and the coumarin components in the outer cortex had a significantly lower ionic strength than those in the phloem.

### Growth and development of the root secretory tract

**Figure 5:**
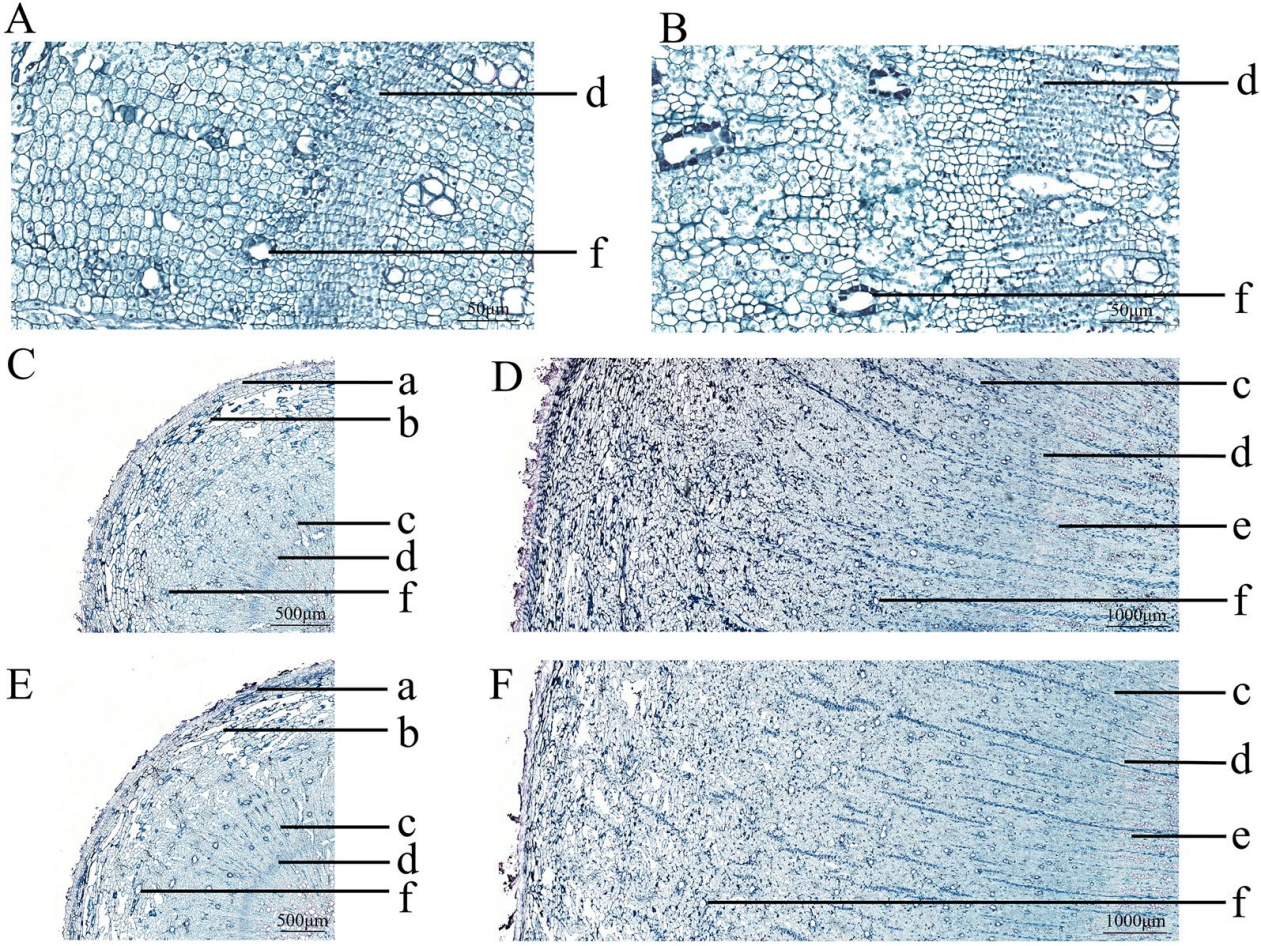
The development process of a secretory tract of HBZ and QBZ (**A** Secretory tract genesis of HBZ; **B** Secretory tract genesis of QBZ; **C** Secretory tract of HBZ in March; **D** Secretory tract of HBZ in July; **E** Secretory tract of QBZ in March; **F** Secretory tract of QBZ in July; a: epiderm; b: cortex; c: phloem; d: cambium; e: xylem; f: secondary canals; Scale bars: A, B: 50 μm; C, E: 500 μm; D, F: 1000 μm).

There are many similarities in the development of root secretory tract between HBZ and QBZ. The primary structural development of the *A. dahurica* root did not result in any secretory canal formation. Therefore, the secretory canals in the *A. dahurica* root should be classified as secondary structures, primarily formed where the cambium's periphery was connected with the secondary phloem (Fig. 5A,B). Secretory canals are more common in the cortex and secondary phloem, their distribution is round, oval, and roughly umbrella-shaped. The image illustrates that the size of secretory canals from the secondary phloem to the outside gradually grows. The number is increasing with the growth and development of the *A. dahurica* root (Fig. 5C–F). After the secondary structure formation in the *A. dahurica* root, the secretory canal initially formed in the secondary phloem. The secretory canal continued to grow outward with the secondary phloem development. During this process, new epithelial cells were constantly embedded into the mature secretory canal epithelial cells, resulting in the gradual expansion of the secretory canal from the secondary phloem to its periphery. Moreover, the expansion of the secondary phloem and the constant formation of new secretory canals contribute to the increase in the number of secretory canals.

### Area and quantity statistics of the root secretory tract

**Figure 6:**
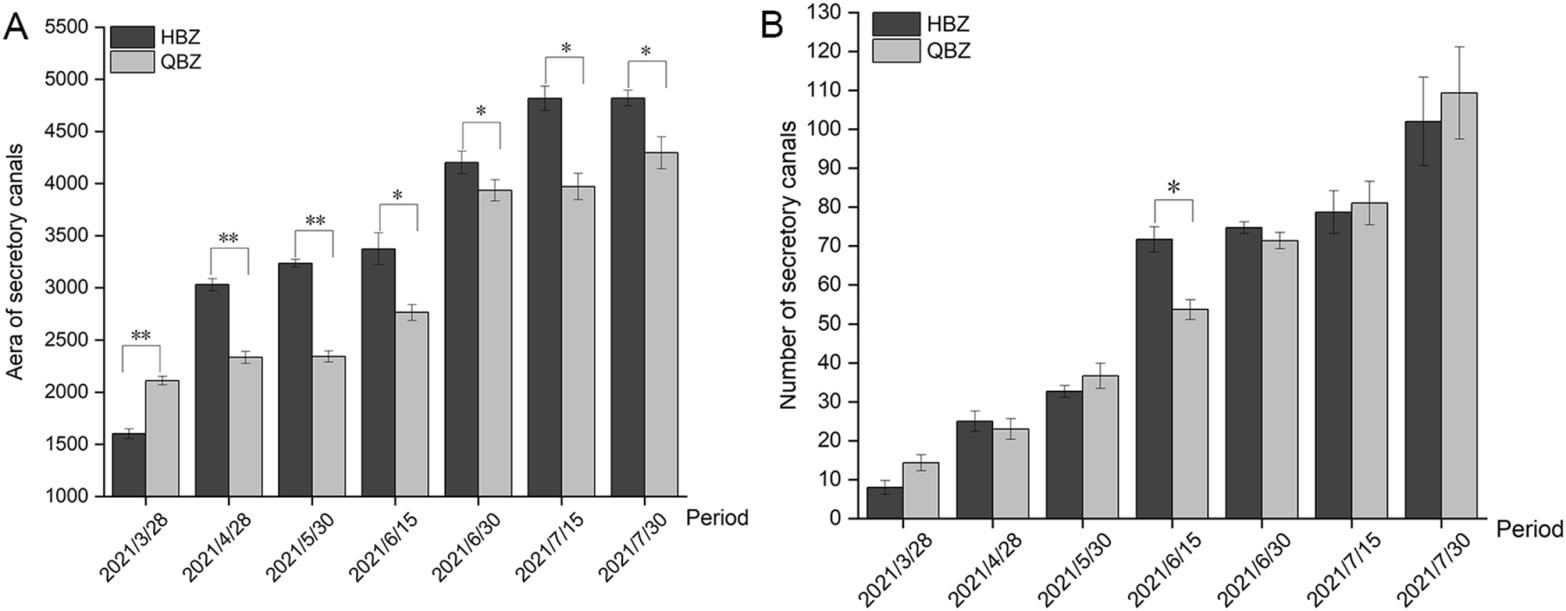
The area and number of root secretory canals of HBZ and QBZ (**A** Area of root secretory canals; **B** Number of root secretory tracts, Data are means ± SD; *p < 0.05; **p < 0.001).

The root cross-section radius, xylem radius, 1/16 of the cortex area, the area of the secretory canal, and the number of secretory canals increased as the root of *A. dahurica* grew, but the above indicators exhibited differences between the two varieties (Table S8, Fig. 6). Specifically, the data measured in HBZ were greater than those measured in QBZ, indicating that the root growth rate of HBZ was generally superior to that of QBZ during the growth and development of *A. dahurica* root. The distribution coefficient of secretory canals was obtained by multiplying the cortex area, the size of secretory canals, and the number of secretory canals in 1/16 of the root cross-sections. The distribution coefficient of secretory canals of the two species exhibited an upward trend with root development, and the amplification was more pronounced as the *A. dahurica* root grew. However, the two varieties of *A. dahurica* still have differences. The distribution coefficient and amplification of the secretory tract of HBZ were greater than those of QBZ, presenting that the cortex area, secretory tract size, and secretory tract growth rate of HBZ were, to a certain extent, faster than those of QBZ.

### Correlation analysis

**Figure 7:**
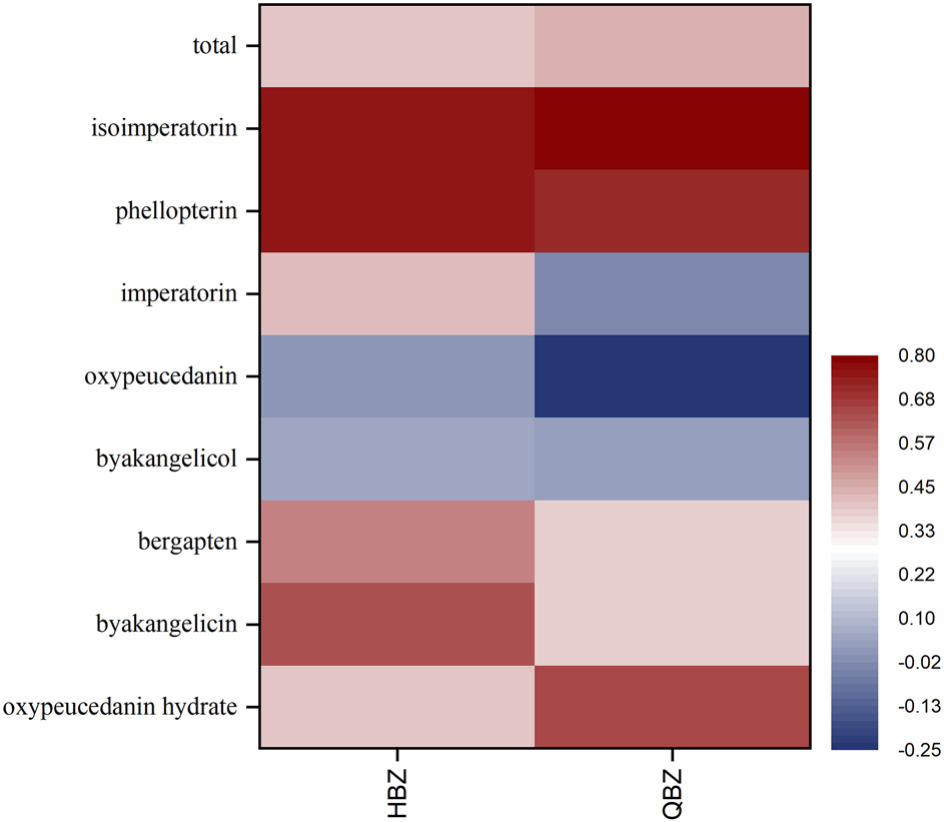
Correlation analysis of root secretory tract and coumarin components in HBZ and QBZ. (The amount of coumarin accumulation is a quantitative value by HPLC).

Figure 7 reveals the correlation between coumarins and distribution coefficient of root secretory tract. The QBZ secretory tract distribution coefficient and oxyimperatorin and imperatorin did not correlate significantly. However, hydrated oxyimperatorin, byakangelicin, bergapten, phellopterin, isoimperatorin, and the total coumarins correlated positively with the distribution coefficients of the secretory tracts in two varieties of *A. dahurica*. There may be differences between the two germplasms in the accumulation of oxyimperatorin and imperatorin, as a quality control component specified in the Chinese Pharmacopoeia, merits further investigation. The study on the localization of coumarin components and root secretory tract indicates that the spatial distribution of coumarin components exhibits a pattern consistent with the localization of the root secretory tract.

## Discussion

Previous studies demonstrated that the yield, imperatorin content, and total coumarin content of HBZ were higher than QBZ under unified cultivation, and genetic coefficient similarity between HBZ and QBZ was low^30,31^. We measured the content of eight coumarin components in different growth stages of HBZ and QBZ under unified cultivation. It was found that there was no significant difference in the types of coumarin components between the two varieties of *A. dahurica* at different growth stages, and the content showed dynamic changes. However, at the period of the highest total coumarin accumulation in each *A. dahurica*, the content of eight coumarin components in HBZ was higher than QBZ. The PCA result presented that the selected coumarin components can represent the quality information of *A. dahurica*, and the comprehensive quality score of HBZ exceeded QBZ, consistent with previous studies, indicating that the germplasm of HBZ was more excellent. This also suggests that the conservation of germplasm resources, breeding improved varieties, and studying suitable ecological factors of HBZ are particularly important.

MALDI-MSI technology has been successfully applied to the visual analysis of coumarins in the *A. dahurica* root, revealing that the coumarins in the primary root are located in the cortex and phloem, primarily concentrated in the phloem^29^. We used DESI-MSI technology to image the coumarins in the root of HBZ and QBZ. The results were consistent with the previous study, indicating the localization of coumarins from HBZ and QBZ in root tissues is generally consistent. This distribution pattern was in accordance with the theory of plant secondary metabolism that plants produce and lay in secondary metabolites, usually toxic to animals in the most vulnerable part (cortex) to resist animals and insects from the outside^32^. Coumarin was distributed in the cortex but not in other parts in late March because the root volume of *A. dahurica* was small, the number of secretory canals was low, and there were only a few secretory canals in the cortex early. Coumarins were distributed in the cortex and enriched in the phloem of the *A. dahurica* root from late April to late July because the volume has expanded, the area and number of secretory canals have increased, and many secretory canals have accumulated in the phloem with the root development.

The correlation study between the root secretory tract of Apiaceae plants and coumarins indicates that the root secretory tract is the accumulation site of coumarin components^33,34^. The research on the correlation between the development of the root secretory canals of *Angelica dahurica* and coumarins mainly focuses on qualitative analysis. Song et al. used paraffin sectioning technology and histochemical localization methods to investigate the accumulation of coumarin components in QBZ. The research results showed that the phloem secretion of QBZ root was the dominant site for the accumulation of coumarins^35^. Li et al. used plant anatomy combined with histochemical localization to dissect and tissue locate the main roots of HBZ, and conducted correlation analysis with the coumarins determined by HPLC. The results indicated that the secretory tract of HBZ was the major accumulation site of coumarins. It initially occurred in the thin-walled tissue of the middle columnar sheath and then occurred in the secondary phloem produced by the division of the middle columnar sheath, which was closely related to the accumulation of imperatorin and isoimperatorin in HBZ^36^. Lan et al. explained for the first time from the perspective of quantitative analysis that the content of imperatorin was positively correlated with the distribution coefficient of the root secretory tract of *A. dahurica*^37^. Our results showed a significant positive correlation between the distribution coefficients of root secretory canals in isoimperatorin and phelloperin, while bergapten, byakangelicin, and oxyeucedanin hydrate had a certain positive correlation with the distribution coefficients of root secretory tracts. However, the exact correlation coefficient between coumarin accumulation and root secretory tract in absolute quantitative analysis needs to be further studied.

DESI-MSI and HPLC were frequently jointly used to analyze the accumulation and distribution of secondary metabolites in TCM^38,39^. HPLC provided accurate quantification, while DESI-MSI provided spatial qualitative analysis. In this study, DESI-MSI can achieve qualitative analysis even without reference materials. Although the DESI-MSI technology cannot accurately quantify the target components, the response ionic strength of different coumarin components in the MSI imaging map can be relatively quantified and matched with the accurate quantitative results of HPLC. DESI-MSI technology detects TCM rapidly due to its simple sample preparation and no matrix assistance. However, factors, such as sample cutting temperature, frozen section embedding agent, and parameter setting, affected imaging results. The premise of ideal imaging is accurate sample preparation methods and reasonable parameters.

The plant secretory structure is a site for exchanging substances and information with the environment, which is affected by environmental variables^19^. Fortuna-Perez et al. discovered that the secretory cavities of plant members of Zornia, a branch of Adesmia, secrete mucus to store water and protect plants from ultraviolet radiation, allowing them to survive in arid and high-UV environments. In contrast, Legu minosae, members of the same family inhabit humid environments, and their secretory cavities contain oil rather than mucus^40^. Kustee et al. reported that the secretory cavities that store mucilage in *I. pes-caprae* (L.) Sweet (Convolvulaceae) and *I. imperati* (Vahl) Griseb enable them to overcome stress caused by high irradiation, high temperatures, and low water availability, allowing their successful establishment in the coastal sand-dune environment^41^. Similarly, the halophytic members of the Plumbaginaceae, Tamaricaceae, and Frankeniaceae families have evolved salt glands to adapt to their arid and saline habitat^42^. Rodríguez-García et al. covered that the resin yield and the number of resin channels positively correlate with temperature and water deficit^43^. In these plants, researchers have been paying attention to adapting secretory structures to their environment. In this study, the results indicated that the location of HBZ and QBZ is identical. However, HBZ has a larger area of root secretory tract than QBZ in the same developmental stage. Climate and soil differences between the north and south may influence the root secretory tract diversity. HBZ and QBZ have separately grown in the south and north of the Qinling Mountains-Huaihe River of China for a long time, respectively, and have formed germplasm resources that adapt to their habitats and can stably inherit^12^. Since ancient times, the Chinese have divided the northern and southern climates and vegetation along the Qinling Mountains-Huaihe River of China. The climate is warm, wet, and rainy, and the cultivated land is mostly wetland in the south, while the climate is dry, cold, and rainless, and the cultivated land is mostly dry in the north. It can be speculated that a warm, humid, and rainy subtropical monsoon climate leads to species diversity in the south. Hence, the root secretory structure in *A. dahurica* accumulates more toxic coumarins and volatile oil to protect it from other species. Ultimately, the secretory structure of HBZ naturally develops better. The effects of specific environmental factors on the secretory structure of *A. dahurica* roots will be studied in subsequent experimental designs to elucidate the mechanism by which this intriguing phenomenon occurs.

## Materials and methods

### Chemicals and reagents

HPLC-grade methanol and acetonitrile were purchased from Fisher Chemical (Pittsburg, PA, USA). HPLC-grade formic acid and anhydrous ethanol were acquired from Chengdu KeLong Chemical Factory (Chengdu, China). Water was purified using a Milli Q water purification system (Water Synergy, Merk Millipore, Germany). Anhydrous Ethanol, Dimethyl benzene, Rhamsan gum, and SafraninO-Fast Green Staining (Servicebio) were bought from Sinopharm Chemical Reagent Co. Ltd. (Chengdu, China). Imperatorin (CHB-201201), isoimperatorin (CHB210110), phellopterin (CHB210106), bergapten (CHB201127), oxypeucedanin (CHB210113), byakangelicol (CHB201227), byakangelicin (CHB210104), and oxypeucedanin hydrate (CHB201220) were obtained from Chengdu Chroma-Biotechnology Co., Ltd. (Chengdu, China). The minimum purity of each of the eight standards was 98.0%.

### Plant materials

**Figure 8:**
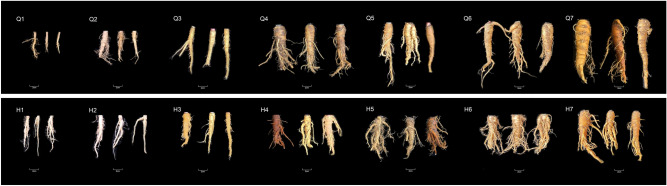
The appearance of *A. dahurica* “Qibaizhi” root and *A. dahurica* “Hangbaizhi” root in seven developmental stages.

*A. dahurica* fresh roots were collected from Chuanbaizhi Scientific Research Demonstration Base, Suining, Sichuan province, identified by Professor Pei Jin from Chengdu University of TCM, and deposited in the State Key Laboratory of Characteristic Chinese Medicine Resources in Southwest China. The nutritional growth stage of *Angelica dahurica* can be divided into the seedling stage, leaf growth peak stage, and root growth peak stage. From January to February, it is in the seedling stage. From March to April, the root system begins to grow, but the growth is slow and mainly concentrated in the aboveground part. From May to July, it enters the root growth peak stage, and the underground part begins to expand, usually harvested in July. Therefore, we set March to July (the period of root growth) as the sampling time. QBZ samples collected during seven different phenological periods from March to July 2021 were numbered Q1, Q2, Q3, Q4, Q5, Q6, and Q7. HBZ samples were numbered from H1 to H7. Each numbered sample had three biological repetitions. All seeds were introduced from the *A. dahurica*’s authentic production area and the main production area, planted under the same environment, and managed using the same cultivation method to exclude the influence of the cultivation environment and cultivation method on the experimental results. Detailed sample information is listed in Table 2 and Fig. 8.

**Table 2:** Detailed sample information including sample number, origin identification, and collection time.

Number	Origin identification	Collection time
Q1	*A. dahurica* cv. ‘Qibaizhi’	2021/3/28
Q2	*A. dahurica* cv. ‘Qibaizhi’	2021/4/30
Q3	*A. dahurica* cv. ‘Qibaizhi’	2021/5/30
Q4	*A. dahurica* cv. ‘Qibaizhi’	2021/6/15
Q5	*A. dahurica* cv. ‘Qibaizhi’	2021/6/30
Q6	*A. dahurica* cv. ‘Qibaizhi’	2021/7/15
Q7	*A. dahurica* cv. ‘Qibaizhi’	2021/7/30
H1	*A. dahurica* cv. ‘Hangbaizhi’	2021/3/28
H2	*A. dahurica* cv. ‘Hangbaizhi’	2021/4/30
H3	*A. dahurica* cv. ‘Hangbaizhi’	2021/5/30
H4	*A. dahurica* cv. ‘Hangbaizhi’	2021/6/15
H5	*A. dahurica* cv. ‘Hangbaizhi’	2021/6/30
H6	*A. dahurica* cv. ‘Hangbaizhi’	2021/7/15
H7	*A. dahurica* cv. ‘Hangbaizhi’	2021/7/30

### Configuration of sample solution and standard solution

Briefly, 0.5 g dried sample powder (water content < 14%) was placed in a 50 mL conical flask with a stopper, followed by 25 mL of a 50% ethanol–water solution ultrasonically dissolved for 1 h. Then, the obtained solution was centrifuged at 1500 rpm for 5 min. The supernatant was injected into the HPLC system after filtering through a 0.22 μm microporous filter membrane. After accurately weighing an appropriate amount of each reference substance and preparing single-component standard solutions with methanol, the mixed standard solution was obtained. Additionally, linearity, precision, stability, repeatability, and recovery tests were performed.

### Chromatographic conditions of HPLC

The samples were analyzed using Ulti Mate 3000 HPLC (Thermo Fisher Scientific, USA). The chromatographic column model was Agilent Zorbax Eclipse XDB-C18 (250 mm × 4.6 mm, 5 μm, Agilent, USA), the mobile phase was 0.1% formic acid–water (A) and acetonitrile (C), and gradient elutes was chosen. The elution program was set as follows: − 3 ~ 0 min, 10% C; 0 ~ 10 min, 10 ~ 25% C; 10 ~ 30 min, 25 ~ 50% C; 30 ~ 43 min, 50 ~ 65% C; and 43 ~ 45 min, 65 ~ 10% C. The volume flow was 1.5 mL/min, the injection volume was 15 µL, the column temperature was 30 °C, and the detection wavelength was 254 nm. The quantitative results of coumarin content were also used in the later correlation analysis with the distribution coefficients of root secretory tracts.

### Sample preparation of DESI-MSI

Transverse sections of *A. dahurica* roots were removed from the − 80 °C refrigerator and thawed for 24 h. A one-eighth sector of the original transverse section area was quickly cut to prepare frozen sections. Frozen samples were fixed axially using the sample holder of the frozen sectioning machine with optimal cutting temperature (OCT) compound as the freezing embedding agent. Cross-sections of 70 μm thickness were obtained at − 20 °C and transferred to indium tin oxides (ITO) conductive glass slides with double-sided adhesive.

### Mass imaging condition

The samples were analyzed using Vanquish ultra-high performance liquid chromatography and Q-Exactive quadrupole-electrostatic field orbit trap high-resolution mass spectrometer in positive ion mode with electrospray ion source. DESI parameters were as follows: scan range: 100–1500 m/z; voltage: spray voltage 4.5 kv, cone voltage 40 v, nitrogen sprayer pressure 0.5 Mpa; solvent: 90% methanol aqueous solution + 1 μL/mL LE; flow rate: 2 μL/min; acquisition spatial resolution: 100 μm; sprayer incidence angle: 90°; droplet collection angle: 10 °C; ion source temperature 150 °C. The data was matched to the mzCloud and Vault databases with a primary and secondary mass deviation of 5 ppm. The images were viewed and processed using HDI v1.5 software (Waters Corporation).

### Slice preparation and observation

The upper part of HBZ and QBZ roots were cut to a length of 1 cm (1 cm from the head of the reed) as the tissue sectioning materials. The tissue samples were fixed with Formaldehyde-acetic acid–ethanol (FAA) fixative, embedded after dehydration and waxing, and 4 μm thick sections were prepared under – 20 °C*.* Then, the slices were placed in warm water at 40 °C to spread, the adhesive tablets were dripped, and the slices were dried. Next, the dewaxed sections were placed onto microscope slides and stained with safranin-fast green. Finally, the slices were placed in xylene transparent for 5 min and sealed with neutral gum. Tissue sections (4 μm) were made as described above. Then, the slices were observed using an optical photomicroscope to focus on the secretory tract development process.

### Calculation of distribution coefficient of secretory tract

Binsong NanoZoomer digital pathological (NDP) section scanner was used to obtain *A. dahurica*’s slice images, whereas CaseViewer software was applied to the statistics of transverse section radius, xylem radius, secretory canal size, and quantity of *A. dahurica* root. The root transverse section radius and the xylem radius were primarily used to calculate the cortex area of 1/16 of the transverse section of the *A. dahurica* root, while the size of secretory canals was calculated using the calculation method of the circular and elliptical areas. The number of secretory canals is the total number within any 1/16 of the cortex area in the transverse section of *A. dahurica*. The distribution coefficient of secretory ducts was obtained by multiplying the cortex area, the size of secretory ducts, and the number of secretory ducts in 1/16 of a transverse section of *A. dahurica* root.

### Data processing and analysis

Statistical analyses (PCA and Person Correlation Coefficient) were performed using Origin 2021 (OriginLab, Hampton, Massachusetts, USA). “Mean ± SD” and Significance analysis were using SPSS 22.0 package (SPSS Inc., Chicago, IL, USA).

### Statement of compliance

The plant materials of this study, HBZ and QBZ, are neither endangered plants nor national key protected wild plants according to the "List of National Key Protected Wild Plants" released by the National Forestry and Grassland Administration. In addition, the plant materials were collected with permission from Sichuan Suining Quantaitang Pharmaceutical Co., Ltd. We implemented that all plant experiments described here comply with relevant institutional, national, and international guidelines and legislation.

## Conclusions

The cultivated Chinese *A. dahurica* has differentiated into HBZ and QBZ based on geographical distribution. Long-term geographical isolation has resulted in different germplasm resource qualities. This study indicated that coumarin content in HBZ is higher than in QBZ. The root secretory tract development is superior to that in QBZ, and the distribution of coumarin is consistent with root secretory tract in both kinds of *A. dahurica*. Hence, it is concluded that *A. dahurica,* with high quality, has better root secretory tract development. The root secretory tract can be a factor in evaluating the quality of *A. dahurica*. Additionally, DESI-MSI technology was used for the first time to elucidate the temporal and spatial distribution of coumarin components in *A. dahurica* root tissues. This study provides a theoretical basis for the quality evaluation and breeding of improved varieties of *A. dahurica*. It also references the DESI-MSI technology used to analyze the metabolic differences of various compounds, including coumarin and volatile oil, in different tissue parts of *A. dahurica*.

### Supplementary Information

Supplementary Information.

## Data Availability

The datasets used and/or analyzed during the current study are available from the corresponding author upon reasonable request.

## Abbreviations

HPLC: High-performance liquid chromatography
MSI: Mass spectrometry imaging
MALDI-MSI: Matrix-assisted laser desorption ionization mass spectrometry imaging
AFAI-MSI: Air flow assisted ionization mass spectrometry imaging
SI-MSI: Secondary ion mass spectrometry imaging
DESI-MSI: Desorption electrospray ionization mass spectrometry imaging
QBZ: *Angelica **Dahurica* Cv. ‘Qibaizhi’
HBZ: *Angelica **Dahurica* Cv. ‘Hangbaizhi’
PCA: Principal component analysis
FAA: Formaldehyde-acetic acid–ethanol
NDP: Nanozoomer digital pathological
OCT: Optimal cutting temperature
ITO: Indium tin oxides
TCM: Traditional Chinese medicine

## References

[1] Li D, Wu L (2017). Coumarins from the roots of *Angelica dahurica* cause anti-allergic inflammation. Exp. Ther. Med..

[2] Huang Y (2021). Effects of imperatorin on apoptosis and synaptic plasticity in vascular dementia rats. Sci. Rep..

[3] Zhao H (2022). The *Angelica dahurica*: A review of traditional uses phytochemistry and pharmacology. Front. Pharmacol..

[4] Commission CP (2015). Pharmacopoeia of the People & Republic of China.

[5] Wang Y (2020). Herb research on *Angelica dahurica* in classic prescriptions. Modern Chin. Med..

[6] Yuan C (1979). Identification and organization of *Angelica dahurica* and its original plants. Chin. Herb. Med. Commun..

[7] Xie, Z. Applying the theory of traditional Chinese medicine varieties to welcome the new century of traditional Chinese medicine quality. *Research and Information on Traditional Chinese Medicine*, 22–23 (1999).

[8] Huang L (1999). Rapd analysis of germplasm resources of *Angelica dahurica*. Chin. J. Chin. Mater. Med..

[9] Wang, N. *et al*. *A Study on the Original Plants of the Traditional Chinese Medicine Angelica dahurica Iv. Discussion On the Original Plants, Cultivation History, and Evolution of Wild Plants Related to Angelica dahurica*, 11–14 (2001). 12776342

[10] Xie C (1989). Special acceptance and exchange meeting of the southern group on the national "seventh five year plan" project "sorting and quality research of commonly used traditional chinese medicine varieties". Tianjin Pharm..

[11] Editorial Committee of Flora of China (1992). Flora Reipublicae Popularis Sinicae.

[12] Huang R (2022). Limited genetic diversity and high differentiation in *Angelica dahurica* resulted from domestication: insights to breeding and conservation. BMC Plant Biol..

[13] Chen L (2019). Content determination and quality evaluation of 9 furacoumarin components in *Angelica dahurica* from different origins. Chin. J. Chin. Mater. Med..

[14] Shi H (2022). Chemical comparison and discrimination of two plant sources of angelicae dahuricae radix, *Angelica dahurica* and *Angelica dahurica* var. formosana, by HPLC-Q/TOF-MS and quantitative analysis of multiple components by a single marker. Phytochem. Anal..

[15] Lan Z (2011). Histochemical localization of coumarins in the root of *Angelica dahurica*. Chin. Med. Herald..

[16] Chen Y, Hu Z, Hu B, Shen X, Yan Y (2015). The relationship between the ultrastructure of the secretory canal and the secretion of volatile oil in the roots of *Angelica dahurica*. Acta Bot. Boreali-Occidentalia Sin..

[17] Wink M (2003). Evolution of secondary metabolites from an ecological and molecular phylogenetic perspective. Phytochemistry..

[18] Fahn A (2000). Structure and function of secretory cells. Adv. Bot. Res..

[19] Roshchina VVRV (1993). The Excretory Function of Higher Plants.

[20] Barbosa SM (2021). Effects of light intensity on the anatomical structure, secretory structures, histochemistry and essential oil composition of *Aeollanthus suaveolens* Mart. Ex. Spreng. (Lamiaceae). Biochem. Syst. Ecol..

[21] Rigling ABHB (2003). Effects of irrigation on diameter growth and vertical resin duct production in pinus sylvestris on dry sites in the Central Alps, Switzerland. For. Ecol. Manage..

[22] Buchberger AR, DeLaney K, Johnson J, Li L (2018). Mass spectrometry imaging: A review of emerging advancements and future insights. Anal. Chem..

[23] Mohana KP, Uma SR, Pradeep T (2019). UPLC and ESI-MS analysis of metabolites of *Rauvolfia tetraphylla* L. and their spatial localization using desorption electrospray ionization (DESI) Mass spectrometric imaging. Phytochemistry..

[24] Jing F (2022). Visualizing the spatial distribution of functional metabolites in forsythia suspensa at different harvest stages by MALDI mass spectrometry imaging. Fitoterapia..

[25] Qin L (2018). Recent advances in matrix-assisted laser desorption/ionisation mass spectrometry imaging (MALDI-MSI) for in situ analysis of endogenous molecules in plants. Phytochem. Anal..

[26] Li Y (2022). Unraveling the mystery of efficacy in Chinese medicine formula: New approaches and technologies for research on pharmacodynamic substances. Arab. J. Chem..

[27] Audinot JN (2021). Highest resolution chemical imaging based on secondary ion mass spectrometry performed on the helium ion microscope. Rep. Prog. Phys..

[28] Takats Z, Wiseman JM, Gologan B, Cooks RG (2004). Mass spectrometry sampling under ambient conditions with desorption electrospray ionization. Science..

[29] Gao H, Li Q (2023). Study on the spatial distribution of coumarins *Inangelica dahurica* root by MALDI-TOF-MSI. Phytochem. Anal..

[30] Wu B (2014). Evaluation of *Angelica dahurica* varieties adapted to cultivation in Jiangsu. Jiangsu Agric. Sci..

[31] Guo D, Ma Y, Lv Q, Wang N, Lu X (2010). Comparative study on the content of imperatorin and HPLC fingerprint of radix *Angelica dahurica* from different places. Chin. Med. Mater..

[32] Piasecka A, Jedrzejczak-Rey N, Bednarek P (2015). Secondary metabolites in plant innate immunity: Conserved function of divergent chemicals. New Phytol..

[33] Chen LL (2019). Tissue-specific metabolite profiling on the different parts of bolting and unbolting *Peucedanum praeruptorum* Dunn. (Qianhu) by laser microdissection combined with Uplc-Q/Tof-Ms and Hplc-Dad. Molecules..

[34] Zhang Y, Chen J, Xu J, Li H (2011). Tissue localization, distribution and fluorescence relative quantitative of coumarins in *Changium smyrnioides* Wolff. Lishizhen Med. Mater. Med. Res..

[35] Song J (2017). Analysis of coumarin accumulation sites in radix *Angelica dahurica* by histochemical technique. Guangdong Chem. Ind..

[36] Li B, Han M, Zhang M, Chen Y (2020). Study on the main accumulation sites and content changes of coumarins during the root development of *Angelica dahurica*. Shanxi Agric. Sci..

[37] Lan Z (2016). The histochemical allocation and quantitative research on coumarins in the fresh root of *Angelica dahurica* Var. Formosana by using high content analysis. Lishizhen Med. Mater. Med. Res..

[38] Chen WJ (2022). Distribution of bioactive compounds in different tissues of paeonia lactiflora roots by DESI-MSI and UPLC. Zhongguo Zhong Yao Za Zhi..

[39] Wang D (2022). Study on the dynamic accumulation and distribution of secondary metabolites in coptis chinensis with different growth years by HPLC and DESI-MSI methods. J. Beijing Univ. Trad. Chin. Med..

[40] Fortuna-Perez AP (2021). Secretory structures of the Adesmia Clade (Leguminosae): Implications for evolutionary adaptation in dry environments. Perspect. Plant Ecol. Evol. Syst..

[41] Kuster VC, Da Silva LC, Meira RMSA, Azevedo AA (2016). Glandular trichomes and laticifers in leaves of ipomoea pes-caprae and I. Imperati (Convolvulaceae) from coastal restinga formation: Structure and histochemistry. Braz. J. Bot..

[42] Caperta AD, Róis AS, Teixeira G, Garcia Caparros P, Flowers TJ (2020). Secretory structures in plants: Lessons from the Plumbaginaceae on their origin, evolution and roles in stress tolerance. Plant Cell Environ..

[43] Rodríguez-García A (2015). Influence of climate variables on resin yield and secretory structures in tapped pinus pinaster ait in Central Spain. Agric. For. Meteorol..

